# Lithium ameliorates lipopolysaccharide-induced microglial activation via inhibition of toll-like receptor 4 expression by activating the PI3K/Akt/FoxO1 pathway

**DOI:** 10.1186/s12974-014-0140-4

**Published:** 2014-08-14

**Authors:** Hongquan Dong, Xiang Zhang, Xiaonan Dai, Shunmei Lu, Bo Gui, Wenjie Jin, Susu Zhang, Shu Zhang, Yanning Qian

**Affiliations:** Department of Anesthesiology, The First Affiliated Hospital of Nanjing Medical University, 300 Guangzhou Road, Nanjing, Jiangsu 210029 China; Nanjing Maternity and Child Health Care Hospital Affiliated to Nanjing Medical University, 123 Tianfei Lane, Nanjing, Jiangsu 210004 China; Clinical Research Center, The First Affiliated Hospital of Nanjing Medical University, Nanjing, 300 Guangzhou Road, Nanjing, Jiangsu 210029 China

**Keywords:** FoxO1, lithium, microglia, PI3K/Akt, TLR4

## Abstract

**Background:**

Lithium, an effective mood stabilizer for the treatment of bipolar disorders, has been recently suggested to have a role in neuroprotection during neurodegenerative diseases. The pathogenesis of neurological disorders often involves the activation of microglia and associated inflammatory processes. Thus, in this study, we aimed to understand the role of lithium in microglial activation and to elucidate the underlying mechanism(s).

**Methods:**

Primary microglial cells were pretreated with lithium and stimulated with lipopolysaccharide (LPS). The cells were assessed regarding the responses of pro-inflammatory cytokines, and the associated signaling pathways were evaluated.

**Results:**

Lithium significantly inhibited LPS-induced microglial activation and pro-inflammatory cytokine production. Further analysis showed that lithium could activate PI3K/Akt signaling. Analyses of the associated signaling pathways demonstrated that the lithium pretreatment led to the suppression of LPS-induced toll-like receptor 4 (TLR4) expressions via the PI3K/Akt/FoxO1 pathway.

**Conclusions:**

This study demonstrates that lithium can inhibit LPS-induced TLR4 expression and microglial activation through the PI3K/Akt/FoxO1 signaling pathway. These results suggest that lithium plays an important role in microglial activation and neuroinflammation-related diseases, which may lead to a new therapeutic strategy for the treatment of neuroinflammation-related disorders.

## Introduction

Microglia, the resident immune cells in the brain, play a pivotal role in the immune surveillance of the central nervous system (CNS). Consequently, these cells are likely to play an important role in either the development of protective immune responses or the progression of damaging inflammation during CNS disease states [1–4]. Activated microglia carry out several immune effector functions typically associated with macrophages. When subjected to abnormal stimulations, such as neurotoxins, neuronal debris, or injury, microglia gradually become activated and produce numerous inflammatory mediators, including tumor necrosis factor-alpha (TNF-α), prostaglandin E2 (PGE2), interleukin-6 (IL-6), nitric oxide (NO), and reactive oxygen species (ROS). Accumulation of these pro-inflammatory and cytotoxic mediators is directly deleterious to the neurons and subsequently induces further activation of microglia, resulting in a vicious cycle [5,6]. Thus, inhibition of the microglial activation and the subsequent inflammatory process may help identify novel therapeutic strategies to eliminate the deleterious effects of microglia [3].

TLR4 is a pattern-recognition receptor (PRR) that recognizes distinct pathogen-associated molecular patterns (PAMPs), such as LPS, a bacterial cell-wall component, and cytokines [7]. After binding to its ligand, TLR4 recruits signaling adaptors and initiates a series of signaling cascades that result in the activation of NF-κB and the release of inflammatory cytokines [8]. Microglial activation can occur through a toll-like receptor (TLR)-4 mediated pathway. The presence of functional TLR4 has shown to have deleterious effects in neurodegenerative and stroke models and plays an integral part in microglial signaling in some disease processes [9].

Lithium, an effective mood stabilizer for the treatment of bipolar disorder, is a neuroprotective and neurotrophic agent efficacious in the treatment of several neurodegenerative conditions [10]. On the other hand, numerous studies have shown that lithium has strong anti-inflammation effects through suppressing the microglial activation and attenuating the overexpression of pro-inflammatory cytokines and chemokines *in vivo* [11]. However, the detailed mechanisms for the anti-inflammation effects of lithium on microglia have not been elucidated. It is unknown whether the anti-inflammation effects of lithium are related to TLR4. In the present study, we investigate the effects of lithium on LPS-induced microglial activation and elucidate the possible mechanisms of its neuroprotective properties.

## Materials and methods

### Reagents

Dulbecco’s modified Eagle’s medium (DMEM), 0.25% Trypsin-EDTA solution and fetal calf serum (FCS) were purchased from Gibco-BRL (Grand Island, NY, USA). Lithium chloride (LiCl) and LPS (Coli 0111:B4) were purchased from Sigma-Aldrich (St. Louis, MO, USA). LY294002, WST-8 dye, RIPA buffer and the BCA kit were purchased from Beyotime (Shanghai, China). The specific mouse anti-rat ED8 (anti-CD11b/CD18) monoclonal antibody (a marker for complement receptor 3 of activated microglia) was purchased from AbD Serotec (Raleigh, NC, USA). Fluoroshield mounting medium with 4,6-diamidino-2-phenylindole (DAPI) was purchased from Abcam (Hongkong, China). Rat IL-6 Immunoassay Kit and Rat TNF-α Immunoassay Kit were obtained from R&D Systems, Inc. (Minneapolis, MN, USA). Rabbit anti-TLR4 polyclonal antibody was purchased from Abcam (Hongkong, China). Specific rabbit monoclonal antibodies against p-PI3K, p-Akt, p-FoxO1, PI3K, Akt, FoxO1 and GAPDH, and secondary anti-rabbit and anti-mouse antibodies were all purchased from Cell Signaling (Boston, MA, USA). A FITC-conjugated goat anti-mouse IgG antibody was purchased from Santa Cruz (Santa Cruz Biotechnology, CA, USA).

### Microglia-enriched cultures

Primary rat microglial cells were prepared as described in a previous protocol, with slight modifications [12]. Briefly, whole brains were isolated from Sprague Dawley (SD) rats at postnatal day one to two. The meninges and blood vessels were removed completely in cold D-Hank’s buffered saline. Next, the brains were minced with sterile scissors and digested with 0.25% Trypsin-EDTA solution for 10 minutes at 37°C. Trypsinization was stopped by adding an equal volume of culture medium, which was high-glucose DMEM containing 10% FBS and penicillin (100 U/ml)/streptomycin (100 μg/ml). The dissociated cells were passed through a 100-μm pore mesh, pelleted at 1,500 rpm for 5 minutes, and resuspended in culture medium. The cells were seeded on poly-D-lysine precoated cell culture flasks and cultured at 37°C in a humidified atmosphere of 5% CO_2_/95% air. The medium was replaced every three to four days after seeding. After the glial cells formed a confluent monolayer (10 to 14 days), the microglial cells were separated from the astrocytes by shaking for 5 hours at 150 rpm and seeded into 6-well culture plates at a density of 10^5^ cells/cm^2^. After 24 hours of culture, the cells were starved overnight and then subjected to treatments. The purity of the microglia was >98% as determined by OX-42 (CD11b)-IR. The cells were pretreated with LiCl or LY294002 for 30 minutes before LPS simulation.

### Cell viability

2-(2-Methoxy-4-nitrophenyl)-3-(4-nitrophenyl)-5-(2,4-disulfophenyl)-2H-tetrazolium Sodium Salt (WST-8) is better than 3-(4,5-dimethyl-2-thiazolyl)-2,5-diphenyl-2-H-tetrazolium bromide (MTT) for analyzing cell viability, because it can be reduced to soluble formazan by dehydrogenase in the mitochondria and has low cytotoxicity. The cell viability was measured using the WST-8 dye according to the manufacturer’s instructions. Briefly, the microglial cells were seeded into 96-well plates at a density of 3 × 10^4^ cells/well. Following this treatment, the WST-8 dye was added to each well, then the cells were incubated at 37°C for 2 hours and the absorbance was determined at 450 nm using a microplate reader.

### TNF-α and IL-6 assay

The amount of TNF-α and IL-6 in the culture medium was measured with a commercial ELISA kit from R&D Systems.

### Western blotting

Cellular proteins were extracted from the primary microglial cells using RIPA buffer. The protein concentration in the supernatant fluid of the lysate was measured using a BCA kit. Proteins (50 μg) in the cell extracts were denatured with sodium dodecyl sulfate (SDS) sample buffer and separated using 10% SDS-polyacrylamide gel electrophoresis (PAGE). The proteins were transferred to a polyvinylidene fluoride (PVDF) microporous membrane (Millipore, Bedford, MA, USA), which was then blocked with 5% skim milk for one hour at room temperature. The membrane was incubated with primary antibody overnight at 4°C. The following primary antibodies were used: rabbit monoclonal anti-TLR4, −p-PI3K (p85α), −p-Akt (ser473), −p-FoxO1, −PI3K, −Akt, −FoxO1, and mouse monoclonal anti-GAPDH (1:1000). After adding the anti-rabbit or anti-mouse secondary antibody (1:1000) for 1 hour, the protein bands on the membranes were detected with an enhanced chemiluminescence kit.

### Immunofluorescence

To determine the activation of the microglia, the cells were fixed with 4% paraformaldehyde for 30 minutes; non-specific binding was blocked by incubating cells in a 5% BSA and 0.1% Triton X-100 solution for 1 hour at room temperature. The microglial cells were incubated with mouse anti-ED8 monoclonal antibody (1:300) in the blocking solution overnight at 4°C. After three washes with PBS, the microglial cells were incubated with the corresponding FITC-conjugated goat anti-mouse IgG (1:200) for 2 hours at room temperature and the nuclei were stained with DAPI. Fluorescent images were acquired using a confocal microscope.

### Flow cytometry analysis

The microglial cells were pelleted by centrifugation at 1,500 rpm for 5 minutes, and then fixed in 4% paraformaldehyde for 30 minutes. After washing, the cells were resuspended in PBS. To determine the microglial activation, the cells were incubated with PE-conjugated mouse anti-rat ED8 monoclonal antibody or isotype control (1:200) at 37°C for 1 hour, resuspended in PBS and analyzed on a FACS Calibur flow cytometer using CellQuest software (BD Biosciences, San Jose, CA, USA).

### RNAi transfection

The primary microglial cells were transfected under serum-free conditions with TLR4 (100 nM), FoxO1 (100 nM) or control siRNA sequences using Lipofectamine 2000 (Invitrogen, Carlsbad, CA, USA), according to the manufacturer’s instructions. Eight hours later, the medium was replaced with regular culture medium containing serum, and the incubation was continued for 48 hours at 37°C before the assays were performed.

### Statistical analysis

All experimental results were from at least three separate experiments. The data were represented as the mean ± s.e.m. The significance of the difference between the control and samples treated with various compounds was determined by one-way ANOVA followed by the post-hoc least significant difference test. The differences were considered significant at P <0.05.

## Results

### Effects of lithium and lipopolysaccharide (LPS) on cell viability in microglia

**Figure 1:**
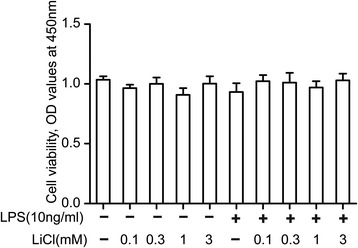
**The effects of lithium and lipopolysaccharide (LPS) on cell viability in primary microglia.** The microglial cells were exposed to different concentrations of LiCl (0.1, 0.3, 1, and 3 mM) and/or LPS (10 ng/ml) for 24 hours. Cell viability was determined using a colorimetric method. Each data point represents the mean ± s.e.m. of at least three separate experiments in which treatments were performed in quadruplicates.

WST-8 assay was used to evaluate the toxic effects of lithium and/or LPS on microglia. The microglial cells were incubated with lithium (0.1, 0.3, 1.0 and 3.0 mM) and/or LPS (10 ng/ml) for 24 hours, and then cell viability was detected by WST-8 assay. Our results indicate that lithium and 10 ng/ml LPS exert no obvious toxic effects on microglia (Figure 1).

### Lithium inhibits lipopolysaccharide (LPS)-induced microglial activation

**Figure 2:**
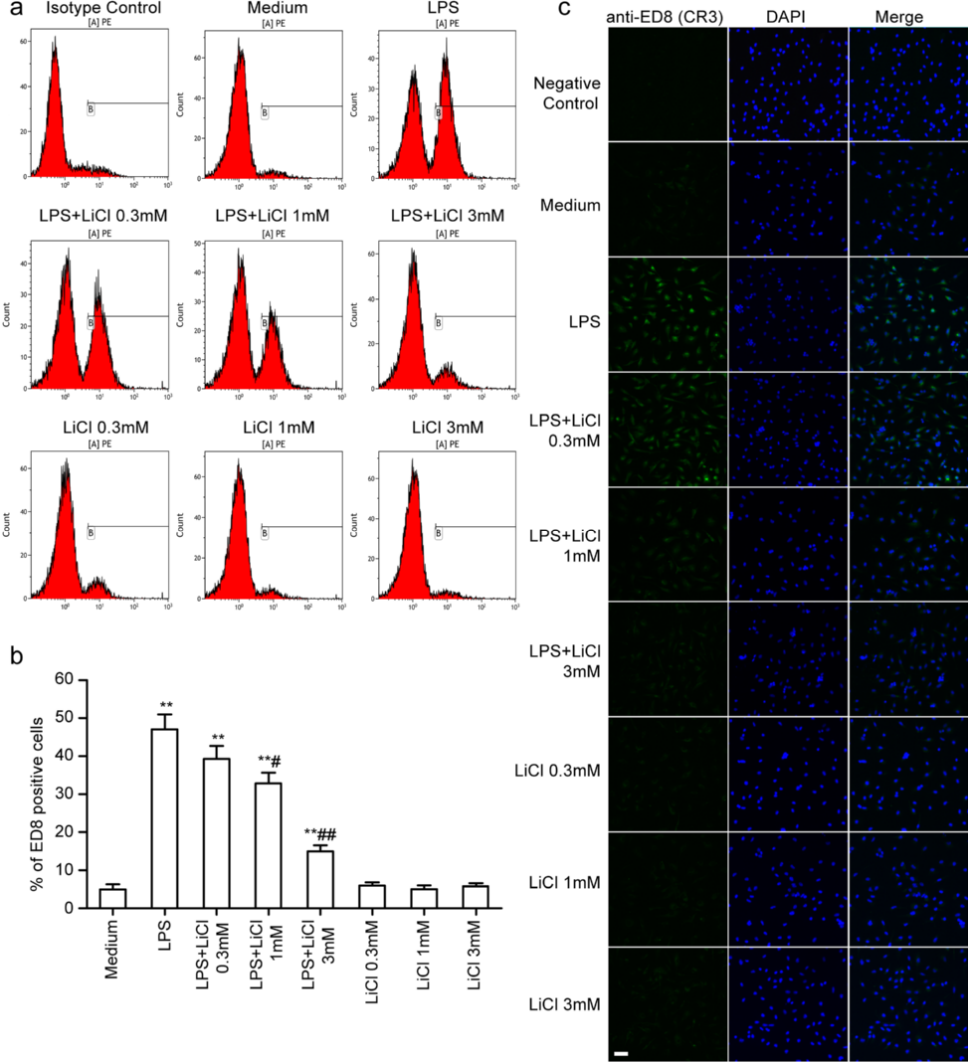
**Lithium inhibits lipopolysaccharide (LPS)-induced microglial activation.** Primary microglial cells were pretreated with LiCl at 0.3, 1 and 3 mM for 30 minutes and then stimulated with LPS at 10 ng/ml for 24 hours. **(a)** and **(b)** For flow cytometric analysis, the cells were incubated with PE-conjugated ED8 antibody at 37°C for 1 hour. **P <0.01 versus the response to medium alone. ^#^ *P* <0.05, ^##^ *P* <0.01 versus LPS treatment groups. The data are presented as the mean ± s.e.m. of three independent experiments. **(c)** The cells were stained with ED8 antibody and upregulated ED8-immunopositive expression (*green*) on the activated microglia was observed using confocal scanning. The blue staining represents DAPI. Scale bar 50 μm.

Activated microglial cells were detected with monoclonal ED8, which recognizes complement receptor 3 (CR3). The microglial cells were pretreated with various concentrations of lithium (0.3, 1.0 and 3.0 mM) for 30 minutes and then stimulated with LPS (10 ng/ml) for 24 hours. Flow cytometric analysis showed that approximately 47.0% microglial cells were activated by LPS, and the number was decreased to 39.3%, 32.9%, and 14.9% when cells were pre-incubated with lithium at 0.3, 1 and 3 mM, respectively (Figure 2a and b). Meanwhile, lithium remarkably inhibited LPS-induced ED8-positive expression (in green) upregulation (Figure 2c). These results suggest that lithium can inhibit LPS-induced microglial activation.

### Lithium downregulates the production of IL-6 and TNF-α by lipopolysaccharide-stimulated microglia

**Figure 3:**
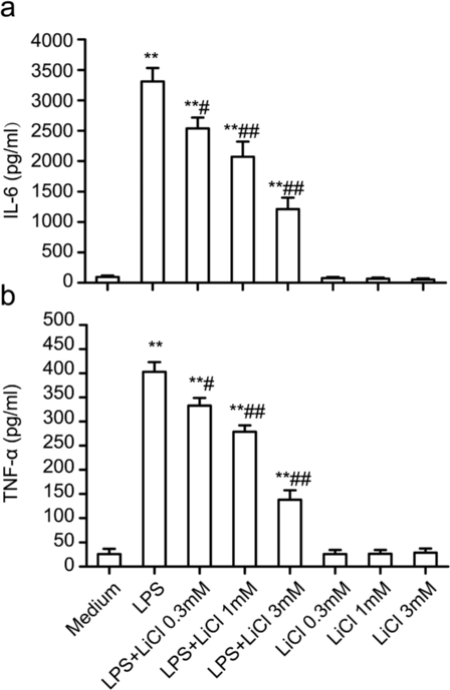
**Lithium downregulates the production of IL-6 and TNF-α** **from lipopolysaccharide (LPS)-stimulated microglia. (a)** and **(b)** Quantification of IL-6 and TNF-α production in media. The primary microglial cells were pretreated with LiCl at 0.3, 1 and 3 mM for 30 minutes and then stimulated with LPS at 10 ng/ml for 24 hours. **P <0.01 versus the response to medium alone. ^#^ *P* <0.05, ^##^ *P* <0.01 versus the LPS treatment groups. The data are presented as the mean ± s.e.m. of three independent experiments.

Microglia-mediated neuroinflammation occurs primarily due to the excessive pro-inflammatory mediators and cytotoxic factors released from activated microglia and their downstream signaling cascades. The levels of pro-inflammatory mediators were determined in the present study. As shown in Figure 3a and b, following the incubation with LPS at 10 ng/ml for 24 hours, the production of IL-6 and TNF-α from primary microglial cells significantly increased by up to approximately 3359% and 1512% that of the control, respectively. Pre-incubation with various concentrations of lithium (0.3, 1.0 and 3.0 mM) for 30 minutes partially abolished LPS-induced IL-6 and TNF-α release in a dose-dependent manner, suggesting that lithium can downregulate LPS-induced production of inflammatory factors.

### TLR4 regulates microglial activation

**Figure 4:**
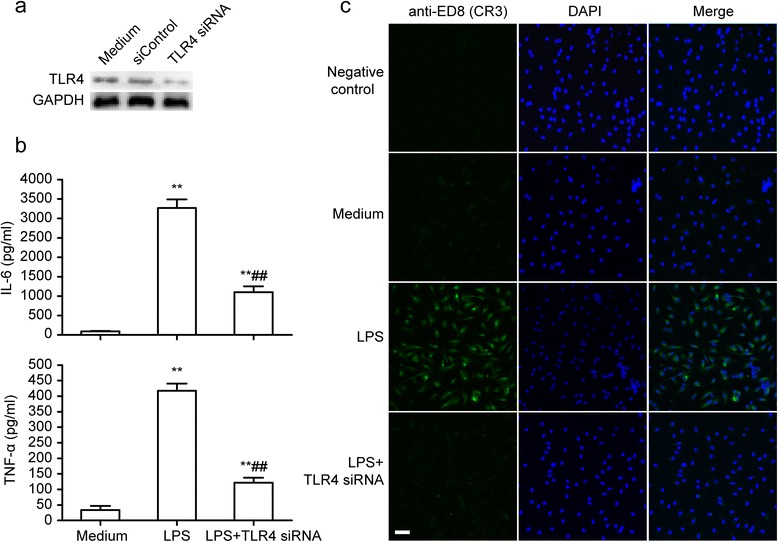
**TLR4 regulates microglial activation.** Primary microglial cells were transfected with TLR4 siRNA (100 nM) or control (siControl) for 48 hours and then stimulated with lipopolysaccharide (LPS) (10 ng/ml) for 24 hours. **(a)** Suppression of TLR4 expression by TLR4 siRNA. **(b)** Quantification of IL-6 and TNF-α production in media, **P <0.01 versus the response to medium alone. ^##^ *P* <0.01 versus LPS treatment groups. The data are presented as the mean ± s.e.m. of three independent experiments. **(c)** The cells were stained with ED8 antibody and upregulated ED8-immunopositive expression (*green*) on activated microglia was observed using confocal. The blue staining represents DAPI. Scale bar 50 μm.

After microglial cells were transfected with TLR4 siRNA for 48 hours, the TLR4-specific siRNA could downregulate 70% of the total TLR4 protein (Figure 4a). The microglial cells were incubated with LPS for 24 hours. As shown in Figure 4b, the TLR4-specific siRNA could inhibit IL-6 and TNF-α release from the LPS-stimulated microglia. Similarly, the ED8-positive expression (in green) was significantly downregulated by the TLR4-specific siRNA (Figure 4c). These results indicate that TLR4 can regulate microglial activation and inflammatory response.

### Effects of lithium on PI3K/Akt/FoxO1 activation and TLR4 expression in microglia

Phosphatidylinositol 3-kinase (PI3K)/Akt is the predominant signaling transduction pathway responsible for the synthesis and production of pro-inflammation mediators and can regulate the expression of TLR4 [13,14]. Our results suggested that lithium could inhibit IL-6 and TNF-α release from the LPS-induced microglia. We investigated whether lithium could affect PI3K/Akt phosphorylation and TLR4 expression in microglia.

**Figure 5:**
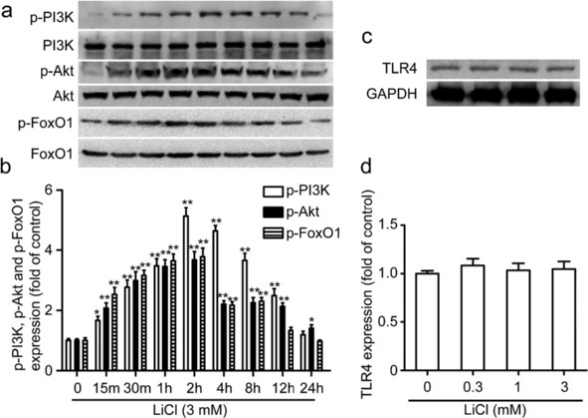
**Effects of lithium on PI3K/Akt activation and TLR4 expression in microglia. (a)** Representative images of western blot for the activation of PI3K, Akt, FoxO1. The primary microglial cells were incubated with LiCl (3 mM) for 15 minutes; 30 minutes; and 1, 2, 4, 8, 12, and 24 hours. **(c)** Effects of lithium on TLR4 expression. The cells were incubated with various concentrations of LiCl for 24 hours. The levels of p-PI3K, p-Akt, p-FoxO1 **(b)** and TLR4 **(d)** were quantified and normalized with their respective total PI3K, Akt, FoxO1 or GAPDH levels. Each value was then expressed relative to the one treated with medium, which was set as 1. *P <0.05, **P <0.01 versus the response to medium alone. The data are presented as the mean ± s.e.m. of three independent experiments.

Treatment with lithium led to a rapid phosphorylation of PI3K, Akt and FoxO1, indicative of PI3K/Akt activation and FoxO1 inhibition, with the peak levels of phosphor-PI3K, phosphor-Akt and phosphor-FoxO1 occurring at 2 h (Figure 5a and b). However, lithium alone had no effect on the expression of TLR4 in our study (Figure 5c and d). These results indicate that lithium can active the PI3K/Akt/FoxO1 signaling pathways in microglia.

### Lithium reduces lipopolysaccharide (LPS)-induced TLR4 expression and inflammatory responses by activating PI3K/Akt signaling

Our data showed that lithium could suppress LPS-induced microglial activation and activate PI3K/Akt signaling. It is known that the PI3K/Akt pathway plays a role in regulating the expression of TLR4 [14]. Thus, we next tested the hypothesis that lithium may regulate TLR4 expression through the PI3K/Akt pathway in LPS-induced microglia.

**Figure 6:**
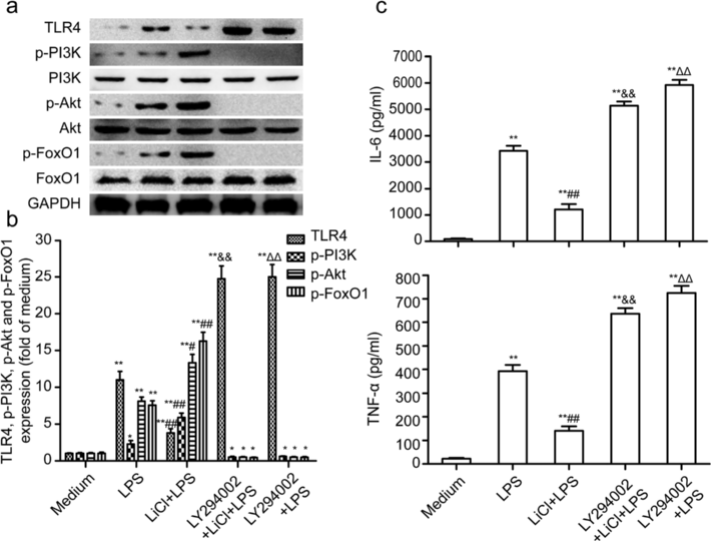
**Lithium reduces lipopolysaccharide (LPS)-induced TLR4 expression and inflammatory responses by activating PI3K/Akt signaling. (a)** Western blotting analysis of TLR4, p-PI3K, p-Akt and p-FoxO1 expression in LPS-stimulated primary microglia after treatment with PI3K inhibitor LY294002 (10 μM) and/or LiCl (3 mM). The levels of p-PI3K, p-Akt, p-FoxO1 and TLR4 **(b)** were quantified and normalized with the total PI3K, Akt, FoxO1 or GAPDH levels, respectively. Each value was then expressed relative to the one treated with medium, which was set as 1.** (c)** ELISA-based IL-6/TNF-α levels in cell culture supernatants. *P <0.05, **P <0.01 versus the response to medium alone. ^#^ *P* <0.05, ^##^ *P* <0.01, ^ΔΔ^ *P* <0.01 versus LPS treatment groups. ^&&^ *P* <0.01 versus LiCl + LPS treatment groups. The data are presented as the mean ± s.e.m. of three independent experiments.

The microglial cells were pretreated with lithium for 30 minutes, followed by the addition of LPS. It indicated that lithium substantially inhibited the TLR4 expression induced by LPS (Figure 6a and b). To investigate the role of PI3K/Akt signaling in the inhibition of TLR4 expression by lithium, we used a PI3K inhibitor (LY294002) to block the PI3K/AKT pathway. The inhibition of PI3K abolished the effect of lithium on the TLR4 expression in LPS-induced microglia (Figure 6a and b). Consistently, the IL-6 and TNF-α production were markedly decreased by lithium, and this effect was abolished by the PI3K inhibitor LY294002 (Figure 6c). As shown in Figure 6a and b, we found that the Akt phosphorylation induced by lithium was abolished by LY294002, suggesting that the lithium-induced Akt activation is mediated by PI3K signaling. Additionally, we found that the inhibition of PI3K could enhance LPS-stimulated TLR4 expression and the production of IL-6 and TNF-α in microglia (Figure 6a, b and c). These results suggest that the PI3K/AKT pathway has a role in LPS-induced microglial activation, and that lithium reduces LPS-induced TLR4 expression and inflammatory responses by activating PI3K/Akt signaling. We also found that lithium could upregulate phosphorylated FoxO1 expression by activating PI3K/Akt signaling in LPS-induced microglial activation (Figure 6a and b).

### Role of FoxO1 in lithium regulates TLR4 signaling

**Figure 7:**
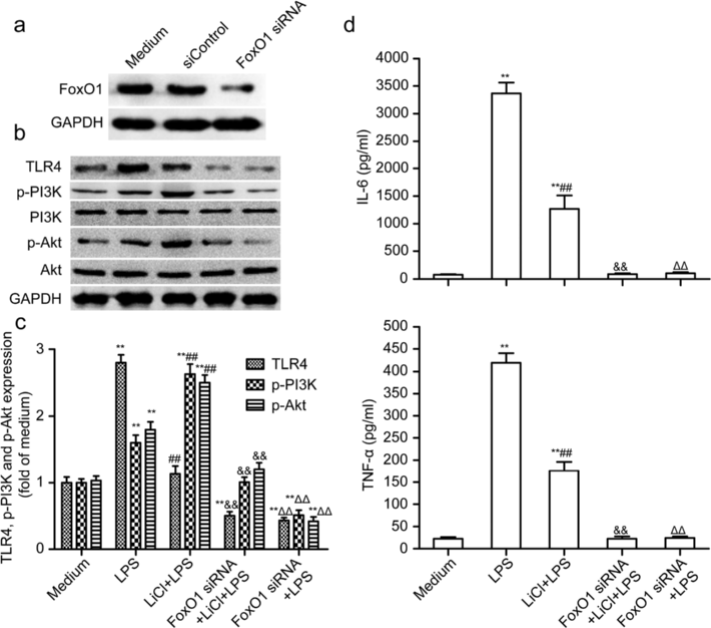
**Role of FoxO1 in lithium regulates TLR4 signaling. (a)** Suppression of FoxO1 expression by FoxO1 siRNA. The primary microglial cells were transfected with TLR4 siRNA (100 nM) or control (siControl) for 48 hours. **(b)** Western blotting analysis of the expression of TLR4, p-PI3K and p-Akt in lipopolysaccharide (LPS)-stimulated primary microglia after transfection with FoxO1 siRNA (100 nM) and/or LiCl (3 mM). The levels of p-PI3K, p-Akt and TLR4 **(c)** were quantified and normalized with their respective total PI3K, Akt or GAPDH levels. Each value was then expressed relative to the one treated with medium, which was set as 1.** (d)** ELISA-based IL-6/TNF-α levels in cell culture supernatants. **P <0.01 versus the response to medium alone. ^##^ *P* <0.01, ^ΔΔ^ *P* <0.01 versus LPS treatment groups. ^&&^ *P* <0.01 versus LiCl + LPS treatment groups. The data are presented as the mean ± s.e.m. of three independent experiments.

The transcriptional activity of FoxO1 is inhibited through its phosphorylation by PI3K/Akt, resulting in its nuclear exclusion and inhibition of target gene expression. To test whether FoxO1 is involved in the effect of lithium on TLR4 expression, the microglial cells were transfected with FoxO1-specific siRNA for 48 hours and then co-stimulated with LiCl and LPS. Our results demonstrated that the transfection of FoxO1-specific siRNA significantly decreased the expression of FoxO1 in the microglia (Figure 7a). As shown in Figure 7b, c and d, the transfection of FoxO1-specific siRNA enhanced the effects of lithium on LPS-induced TLR4 expression, PI3K/AKT phosphorylation and the production of IL-6 and TNF-α. In contrast, the TLR4 protein level induced by LPS was abolished by the FoxO1-specific siRNA (Figure 7b and c). Similarly, LPS-induced IL-6 and TNF-α production was also abolished by the FoxO1-specific siRNA (Figure 7d). These results indicate that FoxO1 is involved in the inhibitor effect of lithium on LPS-simulated TLR4 expression and microglial activation and FoxO1 maintains LPS-triggered TLR4-mediated inflammation signaling.

## Discussion

The anti-inflammatory effects of lithium on neuroinflammation have been widely confirmed and have been associated with the inhibition of microglial activation [11,15]. However, little is known about the mechanism of lithium-mediated inhibition of microglial activation. In this paper, we demonstrate that lithium inhibits LPS-induced upregulation of TLR4 in microglia by activating the PI3K/Akt/FoxO1 signaling pathway.

Microglial activation has been demonstrated to be an early sign that often precedes and triggers neuronal death in chronic neurodegenerative diseases [3,5,16]. Therefore, the inhibition of microglial activation and subsequent neuroinflammation may offer prospective clinical therapeutic benefits for neuroinflammation-related neurodegenerative disorders. Lithium is an established drug used in the treatment of bipolar disorder. Recent *in vivo* studies have indicated that lithium has anti-inflammation properties [11,15], reducing inflammatory response and microglial activation. However, the effects of lithium on microglial activation *in vitro* are not completely elucidated. In this study, we demonstrated that lithium could not only inhibit LPS-stimulated activation of primary microglia, but also reduce IL-6 and TNF-α production from activated microglia.

TLR4, as the receptor of LPS, serves as the primary mediator of innate immune responses to pathogens by activating a cascade of pro-inflammatory events [17]. The activation of TLR4 by LPS triggers signaling via downstream signaling factors such as adaptor myeloid differentiation protein 88 (MyD88), leading to the activation of nuclear factor-κB (NF-κB) and ultimately inducing the expression of inflammation-related genes [18]. Thus, the inhibition of TLR4 can inactivate pro-inflammatory downstream signaling pathways by suppressing differential target gene expression and cellular responses. It is indicated that inhibiting TLR4 expression can suppress LPS-stimulated production of IL-β and TNF-α in the BV-2 microglial cell line [19]. The results presented in our study also demonstrate that the inhibition of TLR4 expression suppresses LPS-induced microglial activation and reduces cytokine production. In addition, we found that lithium inhibited the expression of TLR4, which may be involved in the inhibitory effect of lithium on microglial activation.

PI3K/Akt, which is activated downstream of TLR4 [13,20], is an important regulator of inflammation and is reported to play an important role in negatively regulating LPS-induced IL-6 and TNF-α production from bone marrow macrophages (BMM) [14]. Several recent studies have showed that the neuroprotective effects of lithium is achieved by activating the PI3K/Akt pathway [21,22]. In cultured rat cerebellar granule cells (CGCs), lithium treatment rapidly normalized glutamate-induced inactivation of Akt by activating PI3K and subsequently increasing the phosphorylation of Akt at its Ser473 residue [21]. It is well established that lithium elicits neuroprotection, in part, through its ability to inhibit GSK-3β [23–25]; however, the PI3K-specific inhibitor LY294002 reversed the effects of lithium on GSK-3β [22]. In the present study, we found that lithium could activate the PI3K/Akt pathway in a time-dependent manner in primary microglia. We additionally found that lithium inhibited the expression of TLR4 and cytokine production in LPS-induced microglia by activating the PI3K/Akt pathway, which was abolished by LY294002. The observations suggest that PI3K/Akt is a key signaling effector, which controls LPS-induced microglial activation. These results are in accordance with the fact that PI3K/Akt is a potent suppressor of inflammatory response in monocytes/macrophages [26,27].

FoxO1, also known as FKHR, along with two other mammalian isoforms (FoxO3 and FoxO4), constitute the O class of the forkhead transcription factor family (FoxO), a large array of transcription factors characterized by the presence of a conserved 110-amino acid winged helix DNA-binding domain (DBD) [28]. FoxO1 has important functions in a wide range of cellular processes, such as DNA repair, cell cycle control, stress resistance, apoptosis and metabolism [29–31]. Moreover, the transcriptional activity of FoxO1 is inhibited through phosphorylation by PI3K/Akt, resulting in its nuclear exclusion and inhibition of the target gene expression [29,32]. Mounting studies have showed that FoxO1 plays a key role in hematopoietic and immune cells [33–36]. FoxO1 can stimulate the expression of IL-6, TNF-α and IL-1β in LPS-induced macrophages by upregulating the TLR4 gene expression [37]. However, the function of FoxO1 in neuroinflammation is unclear. In the present study, we found that lithium could upregulate the phosphorylation of FoxO1 by activating PI3K/Akt signaling in LPS-induced microglial activation (Figure 6a and b), indicating that lithium can inhibit the transcriptional activity of FoxO1. To investigate the role of FoxO1 in microglia, we further used FoxO1-specific siRNA to inhibit the expression of FoxO1. We found that the inhibition of the expression of FoxO1 abolished LPS-stimulated TLR4 expression and inflammatory response in microglia, which was even more effective than lithium pretreated microglia. It is suggested that FoxO1, as a transcription factor, can regulate the TLR4 gene expression in microglia, which is in accordance with the fact that FoxO1 is a transcriptional regulator of TLR4 and its pro-inflammatory pathway in macrophages [37]. Being downstream of TLR4, the activation of PI3K/Akt was downregulated with the inhibition of TLR4 expression.

PI3K/Akt, as the downstream of TLR4, its phosphorylation is dependent on the TLR4 activation in LPS-stimulated microglia. Thus, TLR4 is essential for LPS-induced PI3K/Akt activation [38,39]. In Figure 6, as TLR4 was upregulated by LPS, PI3K/Akt was subsequently phosphorylated. PI3K/Akt-mediated phosphorylation of FoxO1 with subsequent nuclear export and degradation is its most important functional regulatory mechanism. Consistent with these findings, we observed that LPS-induced TLR4 activation led to the rapid phosphorylation of FoxO1 (Figure 6a and b). Hence, while FoxO1 maintains LPS-triggered TLR4-mediated inflammation signaling, the TLR4-PI3K/Akt pathway may in turn inactivate FoxO1 transactivation and limit the inflammatory response. This self-limiting feedback mechanism plays a role in prevention of inflammatory response hyperactivation, which could be enhanced by lithium.

**Figure 8:**
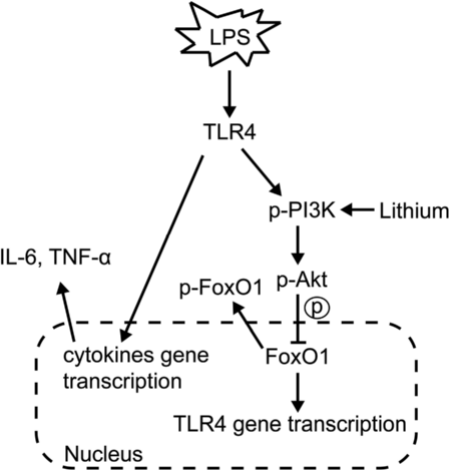
**Schematic illustration of lithium-mediated suppression of neuroinflammation.** FoxO1 maintains lipopolysaccharide (LPS)-triggered TLR4-mediated inflammation signaling. LPS inactivates microglial FoxO1 by inducing PI3K/Akt signaling, which triggers the phosphorylation and nuclear exclusion of FoxO1. The TLR4-PI3K/Akt-FoxO1 axis provides a self-limiting mechanism by which macrophages avoid inappropriate overactivation of inflammation after initiation of the inflammatory response. Lithium, an activator of PI3K/Akt signaling, enhances the self-limiting mechanism and inhibits the activation of microglia. TLR4, toll-like receptor 4; **➞**, lead to/activate; **⊣**, inhibit.

Therefore, we depict putative molecular mechanisms by which lithium may regulate LPS-induced TLR4 expression in microglia (Figure 8). Lithium activates PI3K/Akt signaling, which triggers the phosphorylation of FoxO1. FoxO1, as a transcription factor, can activate TLR4 gene expression. However, once phosphorylated, the phosphorylated FoxO1 proteins are exported from the nucleus and become sequestered in the cytoplasm, which results in the downregulation of TLR4 gene expression.

## Conclusions

In conclusion, this study demonstrates that lithium can inhibit LPS-induced TLR4 expression and microglial activation through the PI3K/Akt/FoxO1 signaling pathway. These results suggest that lithium plays an important role in microglial activation and neuroinflammation-related diseases, which may provide a new therapeutic strategy for the treatment of neuroinflammation-related disorders.

## Abbreviations

CNS: central nervous system
CGCs: cerebellar granule cells
DMEM: Dulbecco’s modified Eagle’s medium
FoxO1: forkhead box O1
IL-6: interleukin 6
LPS: lipopolysaccharide
MTT: 3-(4,5-dimethyl-2-thiazolyl)-2,5-diphenyl-2-H-tetrazolium bromide
NO: nitric oxide
PAGE: polyacrylamide gel electrophoresis
PAMPs: pathogen-associated molecular patterns
PI3K: phosphoinositide 3-kinase
PRR: pattern-recognition receptor
ROS: reactive oxygen species
SDS: sodium dodecyl sulfate
TLR4: toll-like receptor 4
TNF-α: tumor necrosis factor alpha
WST-8: 2-(2-Methoxy-4-nitrophenyl)-3-(4-nitrophenyl)-5-(2,4-disulfophenyl)-2H-tetrazolium Sodium Salt
